# Supplementary material for: Midbrain node for context-specific vocalisation in fish
Source: Nat Commun. 2024 Jan 2;15:189. doi: 10.1038/s41467-023-43794-y (PMC10762186; doi:10.1038/s41467-023-43794-y)
Supplement: Supplementary file 1 — Supplementary Information [file 41467_2023_43794_MOESM1_ESM.pdf]

Supplementary Materials for

Midbrain node for context-specific vocalization in fish

Eric R. Schuppe, Irene Ballagh, Najva Akbari, Wenxuan Fang, Jonathan T. Perelmuter, Caleb H. Radtke, Margaret A. Marchaterre, Andrew H. Bass*

*Corresponding author. Email: ahb3@cornell.edu

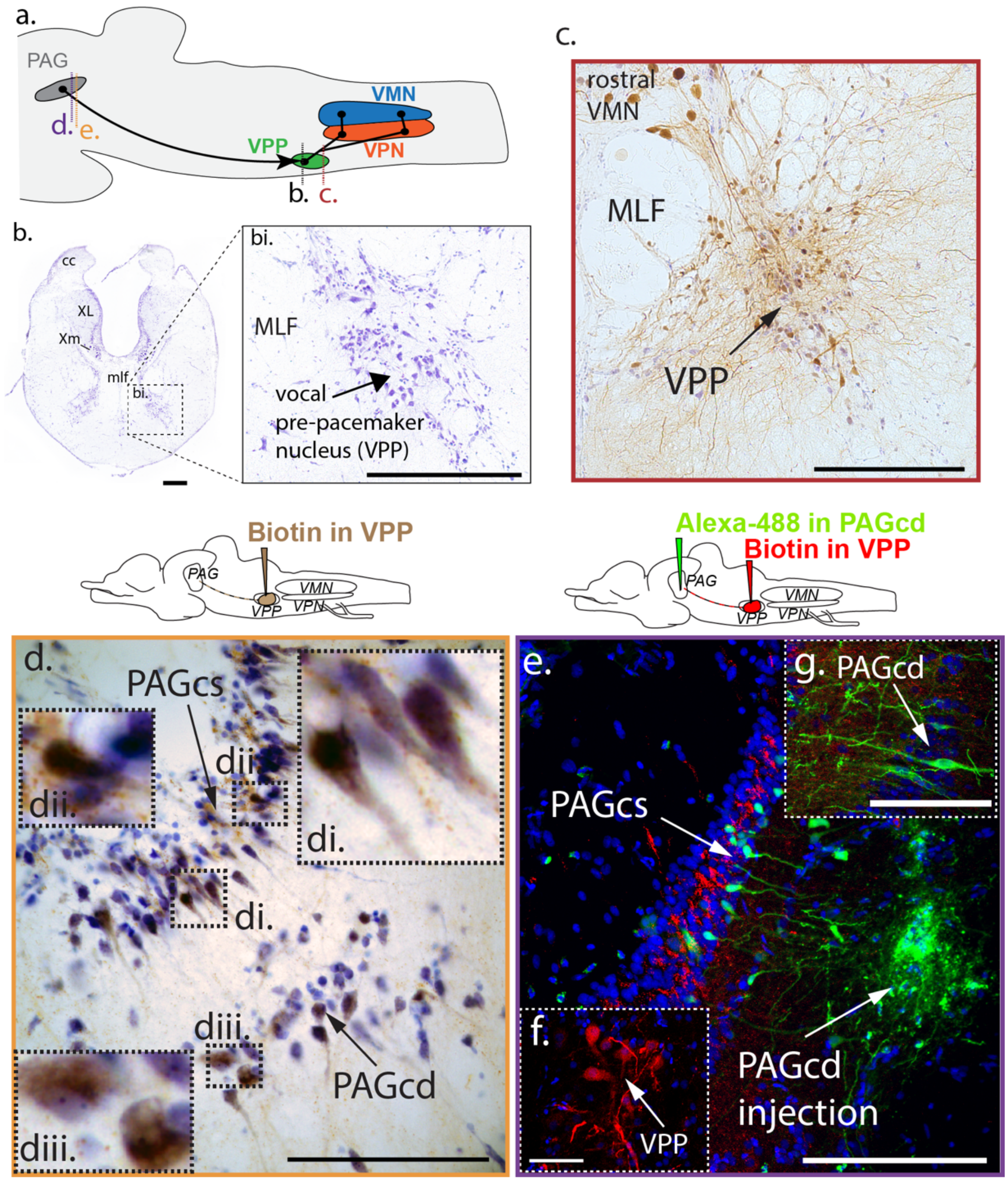

Fig. S1 **(a)** Sagittal drawing illustrating relative locations of (**b, c**) coronal sections through the vocal prepacemaker nucleus (VPP), the main target within the vocal hindbrain circuitry of the lateral periaqueductal gray (PAG)[1–4]. (**b, bi**) Cresyl violet stained section shows cytoarchitecture of hindbrain at the level of VPP. Other abbreviations: cc, crista cerebellaris; mlf, medial longitudinal fasciculus; XL, vagal lobe; Xm, vagal motor nucleus. (**c, d**) Injection of neurobiotin in VPP (**c**) at level of rostral pole of vocal motor nucleus (VMN; brown: neurobiotin, purple: cresyl violet counterstain) led to retrograde labelling of VMN motoneurons and (**d**) somata in the PAG's caudal superficial and deep zones (PAGcs and PAGcd, respectively; n = 3 animals, see sagittal line drawing). (**di-diii**; from regions in corresponding boxes in **d**) Insets illustrate neurobiotin, retrogradely filled cells in both PAGcs (**di, dii**) and PAGcd (**diii**). (**e-g**) Focal injection (n = 2) confined to a vocally active PAGcd site led to filled PAGcs somata with basal processes extending into the PAGcd (**e**), and PAGcd processes extending into the PAGcs and PAGcd (see **g**). This animal also had Alexa-Fluor 594 (red) injected into a vocally active VPP site, resulting in PAG label. (**f**) Representative maximum intensity projection of neurobiotin filled VPP

neurons close to level shown in (**b, bi**). (**g**) Representative example of Alexa-Fluor 488 filled PAGcd neurons (green) ~100 µm caudal to focal injection site in (**e**). Schematic sagittal drawings above (**d**) and (**e**) show relative locations of injection sites. Scale bars are 250 µm (**b-e),** 50 µm (**f**), and 100 µm (**g**).

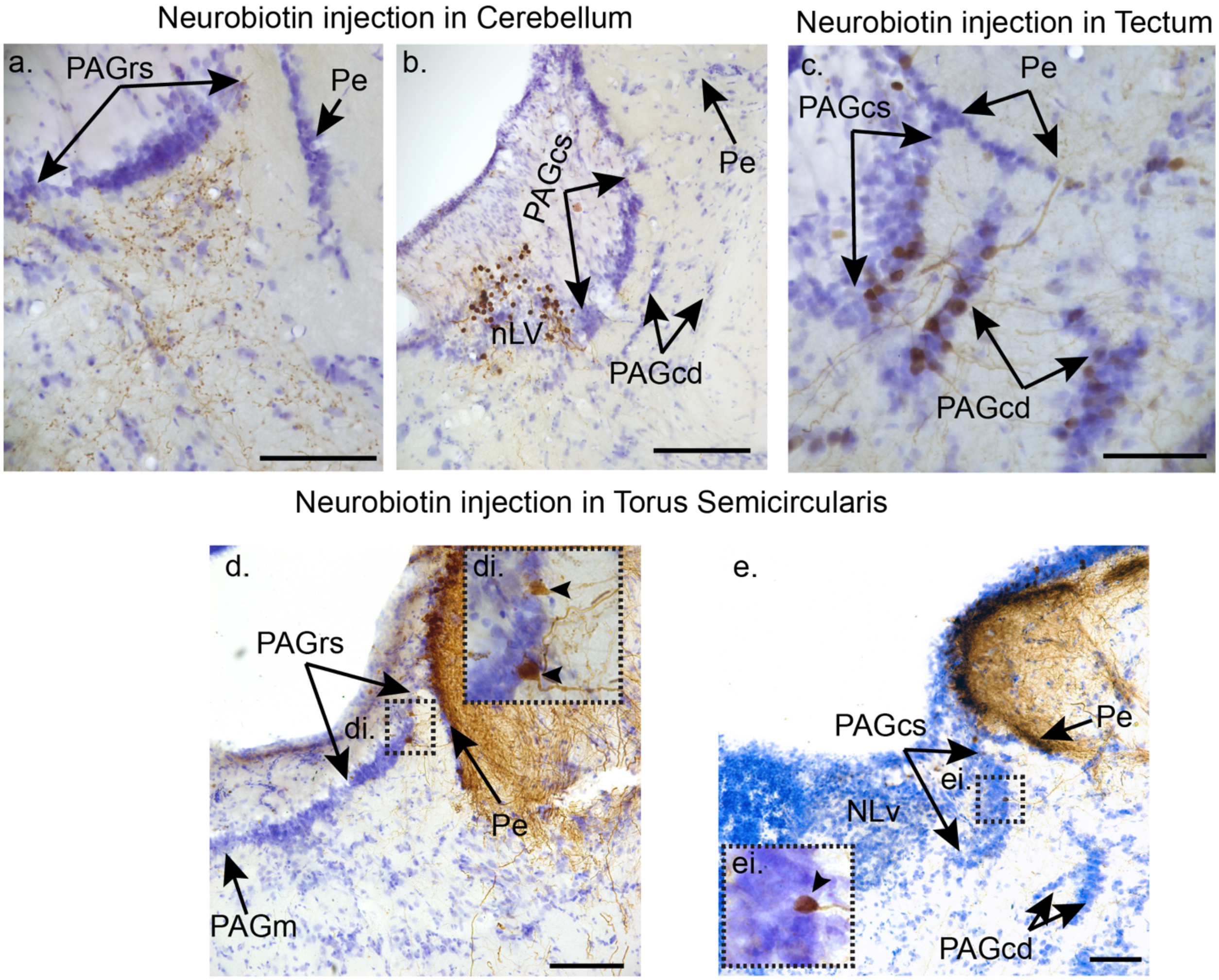

Fig. S2 Different zones of the midbrain periaqueductal gray (PAG) are distinguished by their connections to other brainstem regions. All images from coronal sections counterstained with cresyl violet (purple). (**a, b**) Neurobiotin injections into the corpus of the cerebellum (n = 3 animals) resulted in dense punctate, terminal-like label adjacent to the periventricular cell layer of the rostral superficial zone of the PAG (PAGrs) (**a**), with little label in the superficial and deep zones of the caudal PAG (PAGcs and PAGcd, respectively; **b**). (**c**) Neurobiotin injection into the midbrain tectum (n = 3) yielded ~6-fold or more retrogradely filled cells within the caudal PAG zones compared to the PAGrs. (**d, e**) Coronal sections show retrogradely labelled somata within the far rostral PAGrs (**d**) and far caudal PAGcs (**e**) following neurobiotin injection into auditory division of torus semicircularis (based on material from[55]). (**di, ei**; from regions in corresponding boxes in **d, e)** Shown here are an individual neurobiotin-filled cells in the PAGrs **(di)** and PAGcs (**ei**). All scale bars = 250 µm.

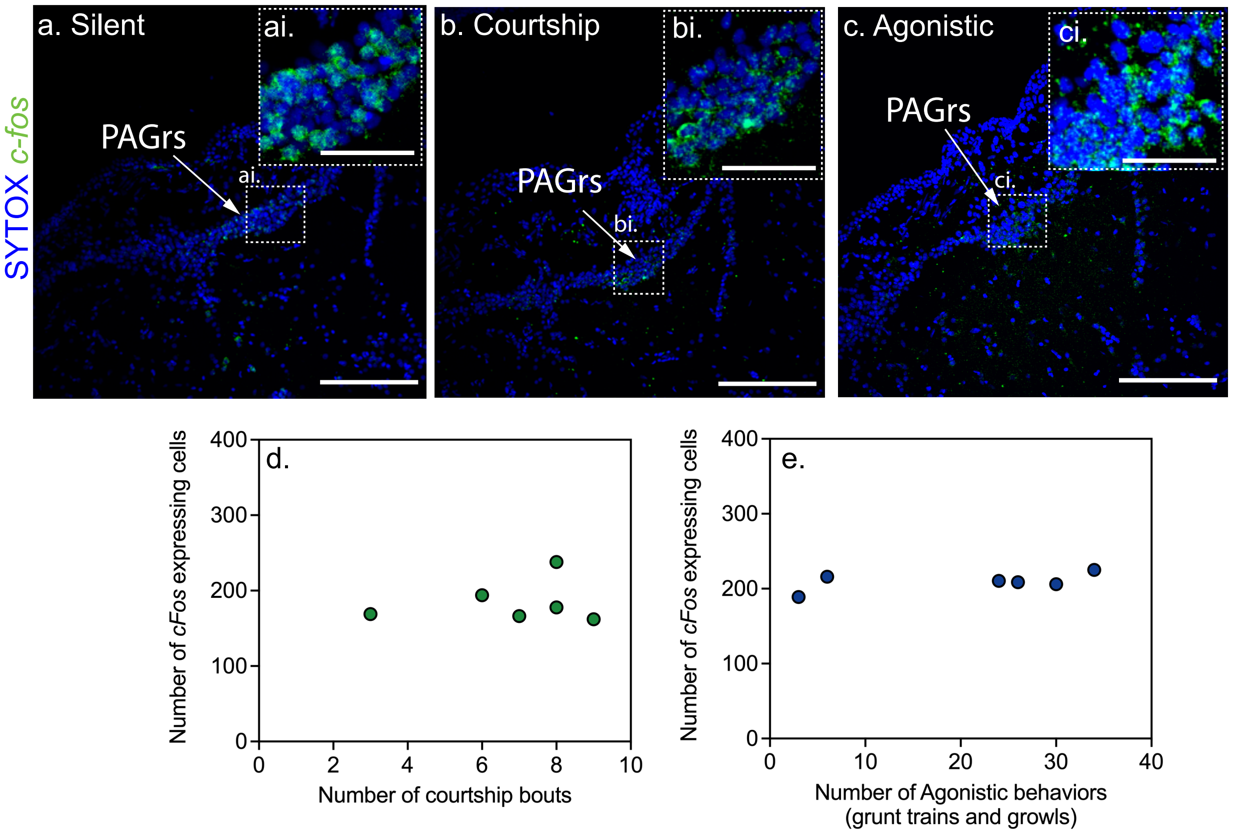

Fig. S3 No differential activation of rostral superficial zone of the midbrain periaqueductal gray (PAGrs) between vocal and silent animals. (**a-c**) Representative maximum intensity projections of confocal images illustrating staining of *c-fos* mRNA (green) in the PAGrs zone of males that were silent (**a**), made courtship hums (**b**), or made agonistic calls when presented with a 3D-printed model of a male at their nest entrance (**c**) during a 40 min trial. Small, hatched white boxes illustrate location of high magnification insets in **ai-ci**. (**d, e**) Plots illustrating no correlation between the number of courtship (**d;** r2 = 0.11, p = 0.47; green-filled circles) or agonistic (**e;** r2 = 0.33, p = 0.23; blue-filled circles) calling bouts and *c-fos* mRNA expression. Scale bars = 250 µm (**a-c**) and 50 µm (**ai-ci**).

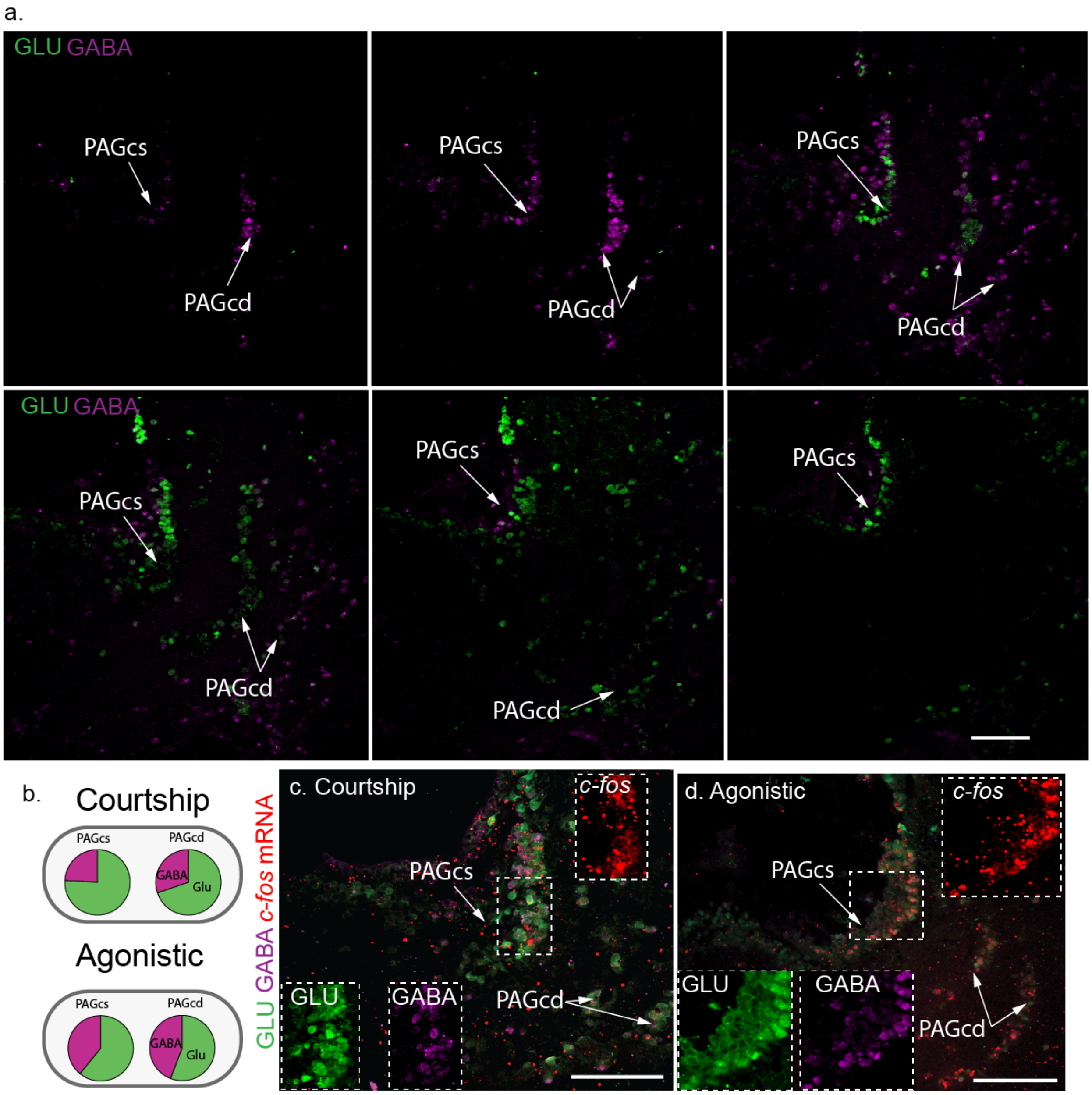

Fig. S4. Differential activation of GABA- and glutamate (GLU)- containing neurons during courtship and agonistic calling.

(**a**) Representative z-stack montage illustrating GLU (green) and GABA (magenta) neurons in the caudal superficial (PAGcs) and deep (PAGcd) zones of the midbrain periaqueductal gray (PAG). (**b**) Graphical breakdown showing relative activation of GLU- versus GABA-containing caudal PAG neurons in courtship and agonistic calling animals. (**c, d**) Representative confocal images of *c-fos* mRNA in GLU- and GABA-containing neurons in the PAGcs (n=5 for both behavioral conditions) and PAGcd (n=5 for both behavioral conditions) of courtship (**c**) and agonistic (**d**) calling males. Single dashed white boxes in PAGcs in (**c, d**) show location of lower left and upper right insets showing representative *c-fos*, GLU, and GABA labeling in each behavioral condition. Scale bars in **a** (bottom right for both rows), **c**, **d** = 250 µm.

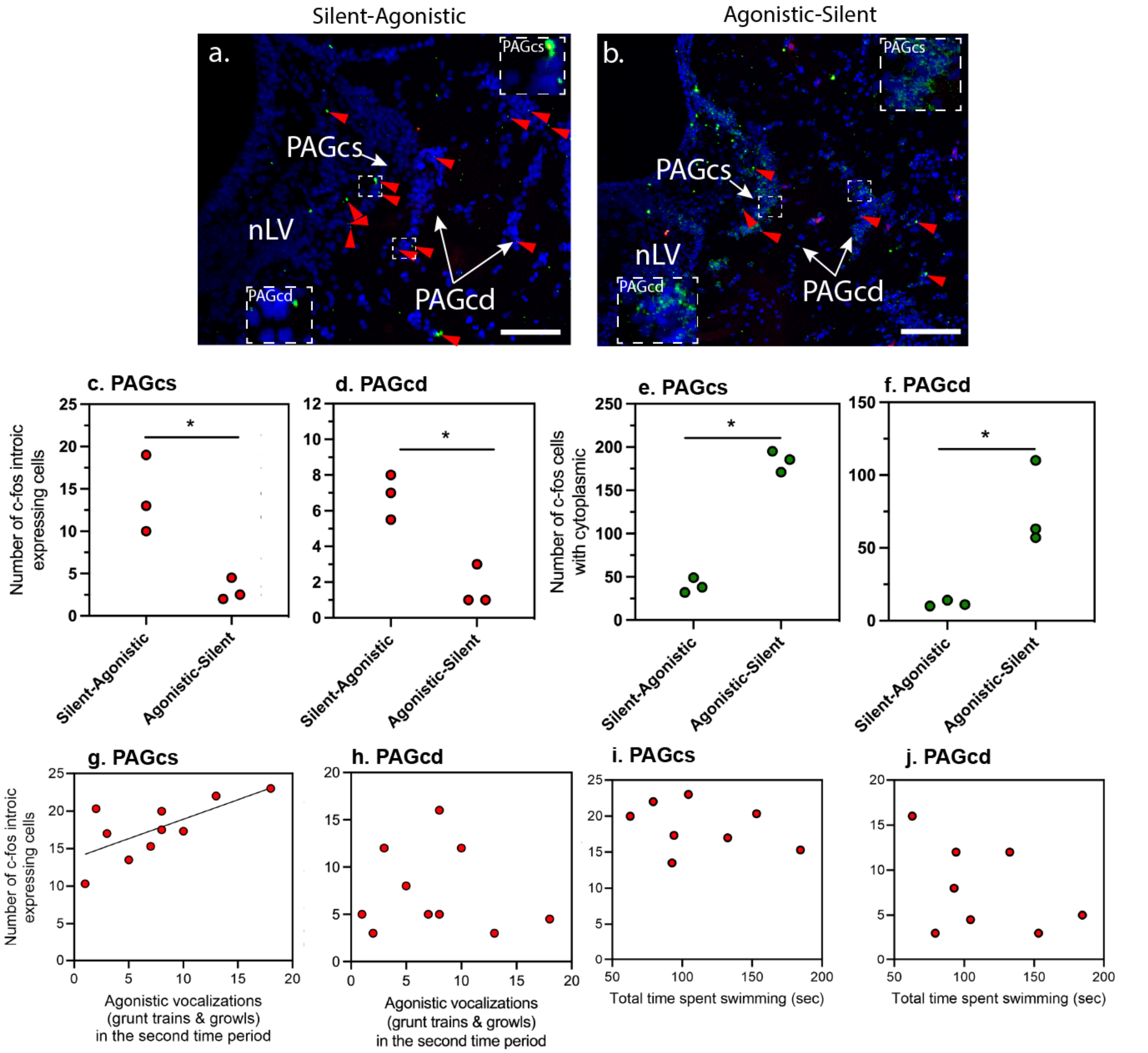

Fig. S5. Time course illustrating the transition from nuclear (silent-agonistic) to cytoplasmic (agonistic-silent) *c-fos* mRNA expression in midbrain periaqueductal gray (PAG).

(**a-f**) To confirm that intronic expression in vocal animals was largely attributed to vocalization, we performed a time course catFISH control (40 min trials). (**a**) Animals (n = 3) that were silent for 35 min and then performed 5 min of agonistic calling exhibited primarily intronic expression (red arrowheads). Lower left and top right insets show higher magnification views of regions outlined in small hatched boxes in the caudal superficial and deep zones of the PAG (PAGcs and PAGcd, respectively). The number of intronic expressing cells was significantly greater compared to animals that performed 5 min of agonistic calling followed by 35 min of silence (**c, d;** PAGcs: $t(5) = 5.27$, $p=0.006$; PAGcd: $t(5) = 4.50$, $p = 0.01$**).** (**b**) Animals (n = 3) that first produced agonistic calls exhibited primarily cytoplasmic expression. Lower left and top right insets show higher magnification views of regions outlined in small hatched boxes in the PAGcs and PAGcd. Intronic expression (red arrowheads) in the PAGcs rarely

overlapped cytoplasmic signal. These animals had significantly higher cytoplasmic *c-fos* mRNA expression (**e, f**; PAGcs: $t(5) = 12.06$, $p=0.0003$; $t(5) = 8.01$, $p=0.001$). Together, these controls confirmed intronic expression observed in vocal animals was primarily a result of the animal producing robust vocal behaviour. (**g, h**) As with *c-fos* mRNA (Fig. 2k), intronic expression was also correlated with the number of agonistic calls in the PAGcs ($r2 = 0.45$, $p = 0.03$), but not the PAGcd ($r2 = 0.001$, $p = 0.93$). (**i, j**) The total amount of time spent swimming during the second 5 min behavioural period of agonistic calling did not correlate with *c-fos* intronic expression in either the PAGcs (**i**; $r2 = 0.08$, $p = 0.48$) or PAGcd (**j**; $r2 = 0.16$, $p = 0.32$).

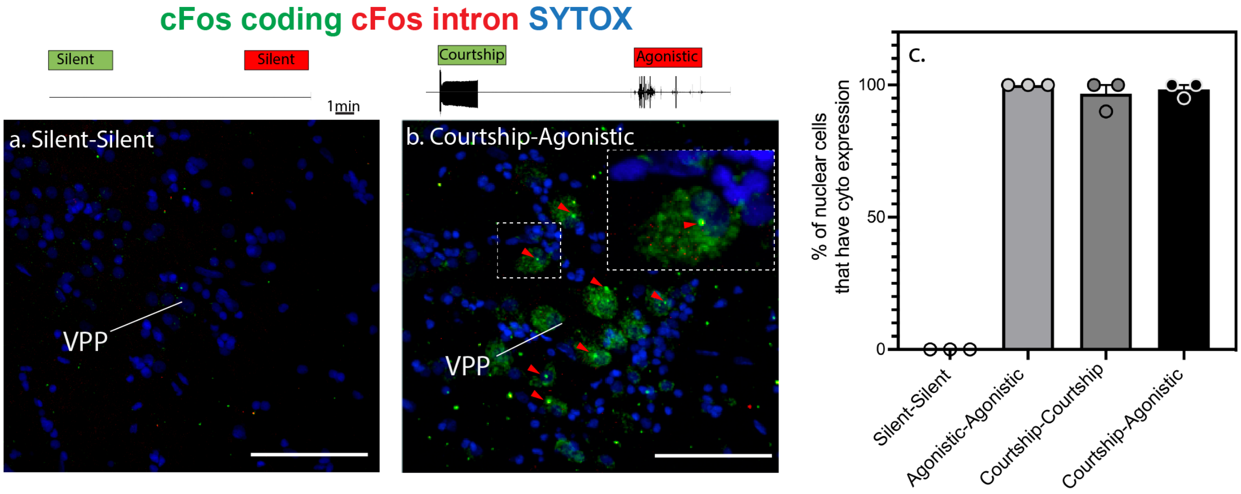

Fig. S6 Call-specific activation in hindbrain vocal prepacemaker nucleus (VPP).
(**a, b**) Representative confocal maximum intensity projections of VPP in silent controls (**a**, n = 3 animals) or fish that made courtship hum calls followed by agonistic calls during two 5 min behavioural periods separated by 30 min of silence (**b**, n = 3). Green signal is *c-fos* mRNA (cytoplasmic, *c-fos* coding), red signal is *c-fos* intron (nuclear expression, red arrowheads), and blue signal is SYTOX deep red. Inset to the right shows high magnification view of small hatched box. (**c**) Nearly all (>90%) VPP neurons expressed both *c-fos* cytoplasmic and nuclear signals during two 5 min behavioural periods separated by 30 min of only agonistic calling, only courtship humming, or courtship humming followed by agonistic calling ($F(3,12) = 696.7$ $p = 5.193e-10$; see Fig. 3 for PAG).

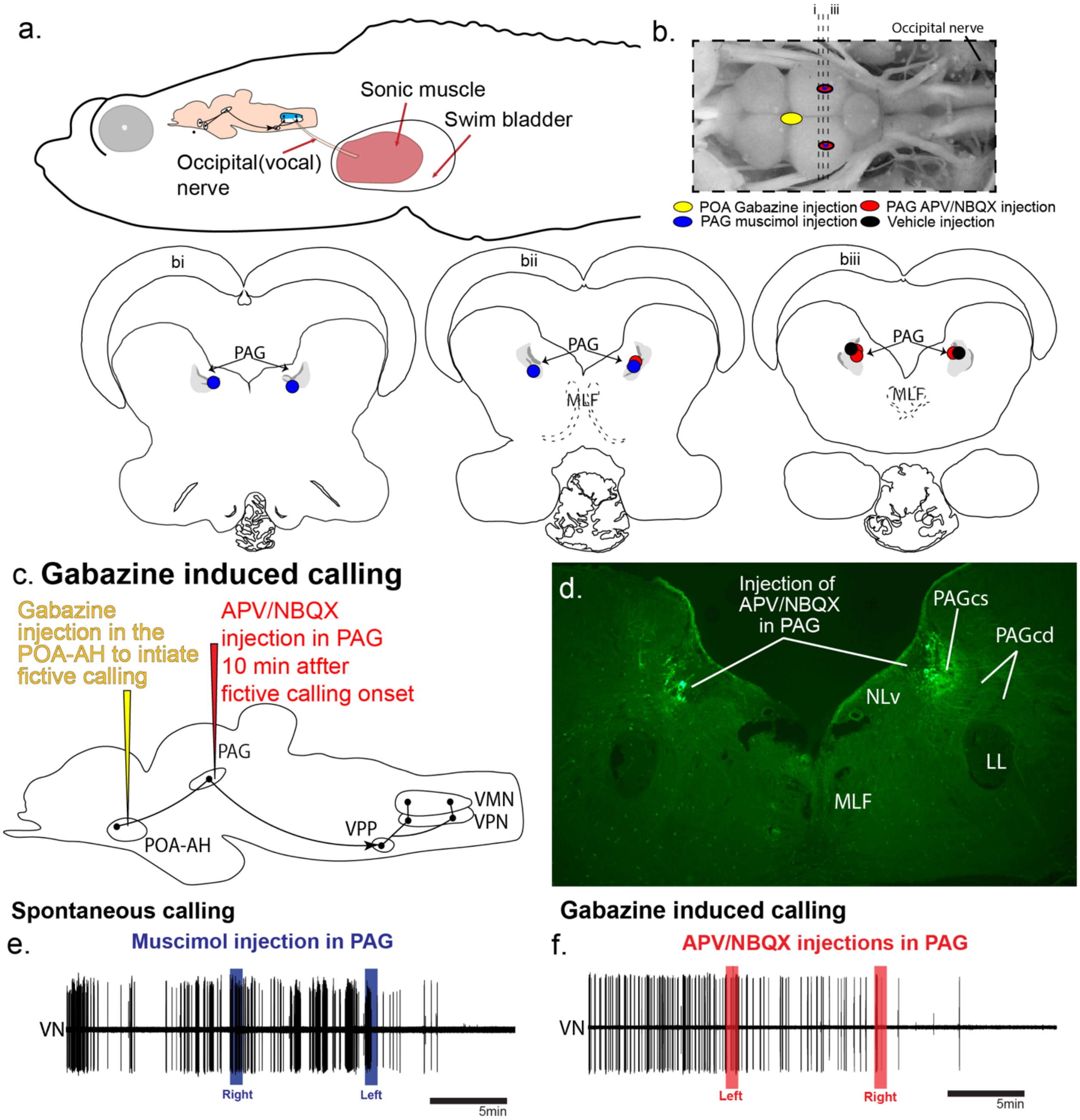

Fig. S7 Vocal system and pharmacological manipulation of the midbrain periaqueductal gray (PAG) vocal region.

(**a**) Line drawing of sagittal view of plainfin midshipman male (*Porichthys notatus*) showing relative location of the brain, including hindbrain vocal motor (blue oval), pacemaker (white oval), and prepacemaker (more rostral small white circle) nuclei. Vocal motor axons give rise to paired vocal occipital nerve roots (proposed homologues of hypoglossal nerve roots[6]), which form a vocal nerve that innervates the ipsilateral sonic muscle attached to the wall of the swim bladder. The paired vocal nerves fire in synchrony, which leads to simultaneous muscle contraction and production of one sound pulse[7]. Multiple pulses form a single sound[7]. Highly synchronous VMN output results in a rapid, temporally precise series of brief compound potentials readily recorded as a vocal motor volley from the occipital

nerve roots and designated a fictive call because it mimics natural call properties[4,7,8]. (**b**) Image of the midshipman brain showing different relative locations of injection sites in the preoptic area-anterior hypothalamus (POA-AH) for gabazine ($GABA_A$ receptor (R) antagonist, yellow, n = 2 animals), and in the PAG for muscimol ($GABA_AR$ agonist, blue, n = 2), a glutamate receptor (GLU-R) antagonist cocktail (APV+NBQX, block AMPA and N-methyl-D-aspartate receptors, respectively; red, n = 2), or a vehicle control (dye + buffer; black, n=1). (**bi-biii**) Illustrations of the relative locations of each injection mapped onto coronal line drawings through the midshipman PAG from rostral to caudal (left to right). (**c**) Sagittal illustration of the midshipman brain depicting the relative injection location of gabazine (yellow) in the POA-AH and bilateral GLU-R antagonists in the PAG to initiate and then silence fictive vocalizations, respectively. (**d**) Micrograph illustrating the location of GLU-R blocker injection (green, Alexa-Fluor 488 mixed with cocktail). (**e, f**) Representative examples of neurophysiological recordings of fictive calls from the vocal nerve (occipital nerve root) showing the effects of muscimol or GLU-R antagonist. In muscimol injected animals, fictive calling rate was reduced completely or partially (average:76.5%, range: 100% - 53%) at 5 min after the second PAG injection on the left side of the brain. Injection of gabazine partially and then almost completely (range: 90% - 97%) silenced fictive calling after injections on the right and then left sides of the brain, respectively. Vehicle control exhibited no decrease, but rather a small increase relative to baseline (115%).

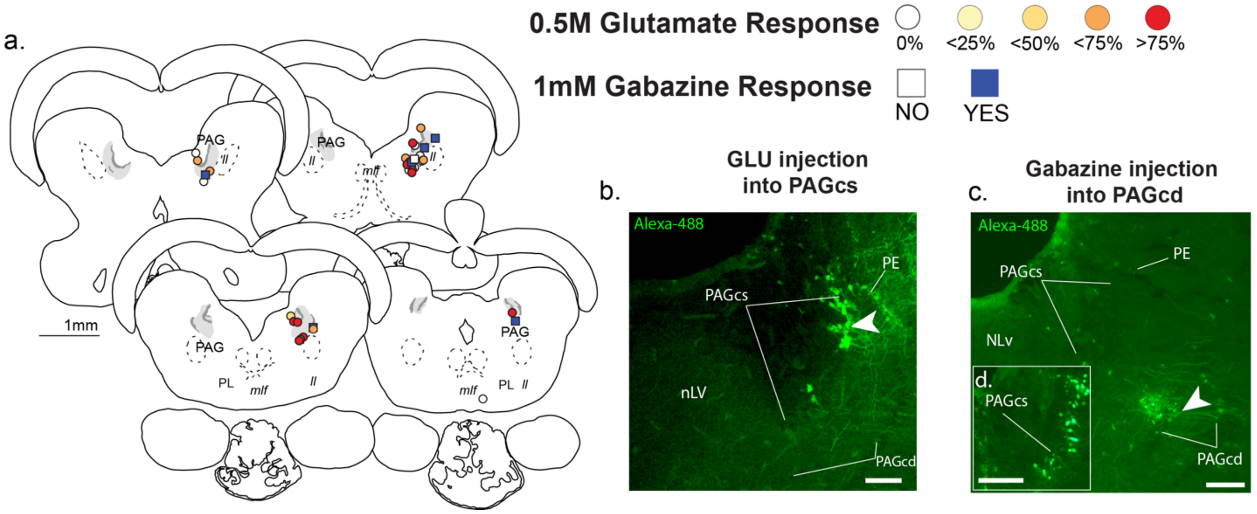

Fig. S8 Injection sites for glutamate (GLU) and gabazine effects on fictive calling

(**a**) Illustrations of the relative locations of each midbrain injection site for GLU and gabazine that evoked fictive calls mapped onto coronal line drawings through the midshipman periaqueductal gray (PAG). Yellow to red filled circles indicate increasing responsiveness to GLU (e.g., a particular site produced fictive calls in multiple trials). Blue-filled squares indicate sites where gabazine induced fictive calls. White-filled circles and squares indicate non-responsive GLU or gabazine sites, respectively. (**b, c**) Representative examples of animal co-injected with GLU and Alexa-Fluor 488 (green) into the PAG's caudal superficial zone (PAGcs, **b**) and caudal deep zone (PAGcd, **c**; dye injection is a different animal than illustrated in Fig. S1e). Analysis of consecutive sections confirmed that the dye was confined to each PAG zone. (**d**) Inset shows labelled somata in PAGcs in a section ~75 um from the focal PAGcd injection site, consistent with images shown in Fig. S1e.

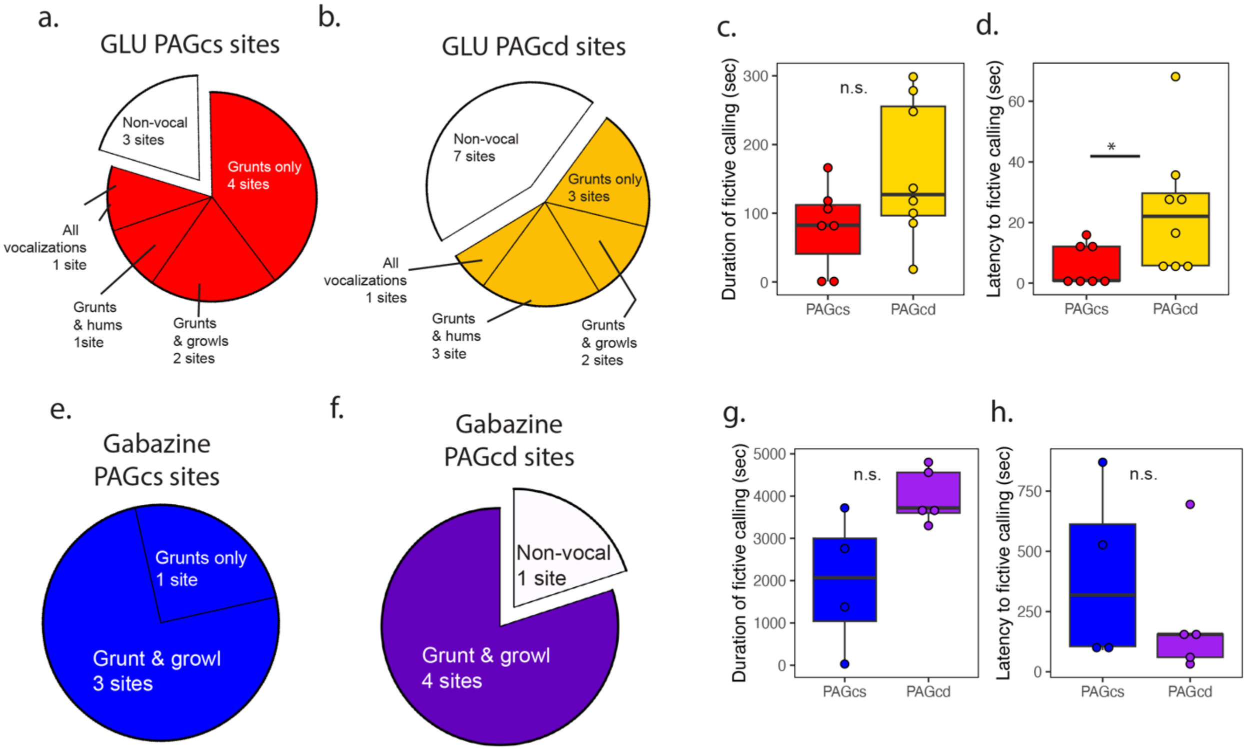

Fig. S9. Types of calls evoked for each caudal periaqueductal gray (PAG) zone following administration of glutamate (GLU) or gabazine.

(**a, b**) Graphical breakdown illustrating the proportion of non-vocal (white) and vocal responses following microinjection of GLU centered in the caudal superficial PAG(PAGcs; red) or deep (PAGcd; yellow) zones of the PAG. (**c, d**) Total duration (t(13)= 1.67, p=0.11) and call latency for fictive calling bouts following injection of GLU ((t(13)= 2.68, p=0.01) into the PAGcs (n=7) or PAGcd (n=8). (**e**, **f**) Graphic breakdown of the non-vocal (white) and vocal responses following microinjection of gabazine into the PAGcs (**e**, blue) or PAGcd (**f**, purple). (**g, h**) Total duration ((t(6)=0.932, p=0.38) and call latency for fictive call bouts following injection of gabazine ((t(7)=1.616, p=0.14 into the PAGcs (blue; n=4) or PAGcd (purple; n=5). Circles on box plots = individual data points n.s. indicates no significant difference. Box plots: center line indicates median edges represent first and third quartiles; whiskers extend to span a 1.5 interquartile range from edges; individual dots are points falling outside range.

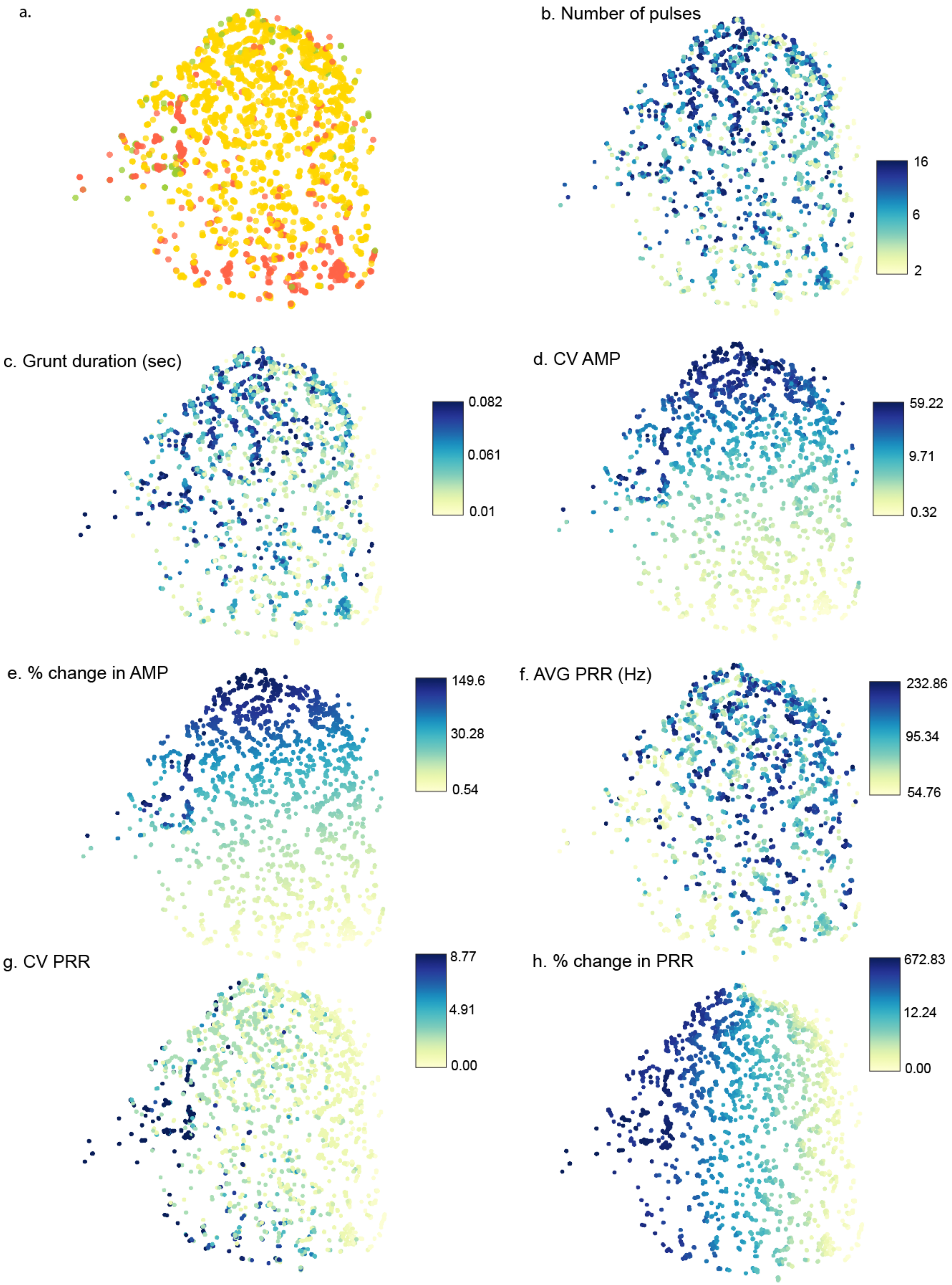
a.
b. Number of pulses
16
6
2
c. Grunt duration (sec)
0.082
0.061
0.01
d. CV AMP
59.22
9.71
0.32
e. % change in AMP
149.6
30.28
0.54
f. AVG PRR (Hz)
232.86
95.34
54.76
g. CV PRR
8.77
4.91
0.00
h. % change in PRR
672.83
12.24
0.00

Fig. S10 Acoustic features contributing to Uniform Manifold Approximation and Projection (UMAP) cluster for glutamate (GLU)-evoked fictive grunts.
Graphical breakdown showing how different acoustic features contribute to UMAP cluster for fictive grunts evoked from GLU injections into the caudal superficial and deep zones of the periaqueductal gray (PAGcs and PAGcd, respectively). (**a**) UMAP plot for fictive (PAGcs, red; PAGcd, yellow) and natural (green) grunts. (**b-h**) UMAP plots showing how all variables measured contribute to each cluster, including grunt pulse number (**b**), duration (sec; **c**) coefficient of variation in amplitude (CV AMP, **d**), percent (%) change in AMP (**e**), average pulse repetition rate (AVG PRR, Hz; **f**), CV in PRR (Hz; **g**), % change in PRR (Hz; **h**). In all plots, yellow is the lowest value and dark blue is the highest value (see key to the right of each graph for details).

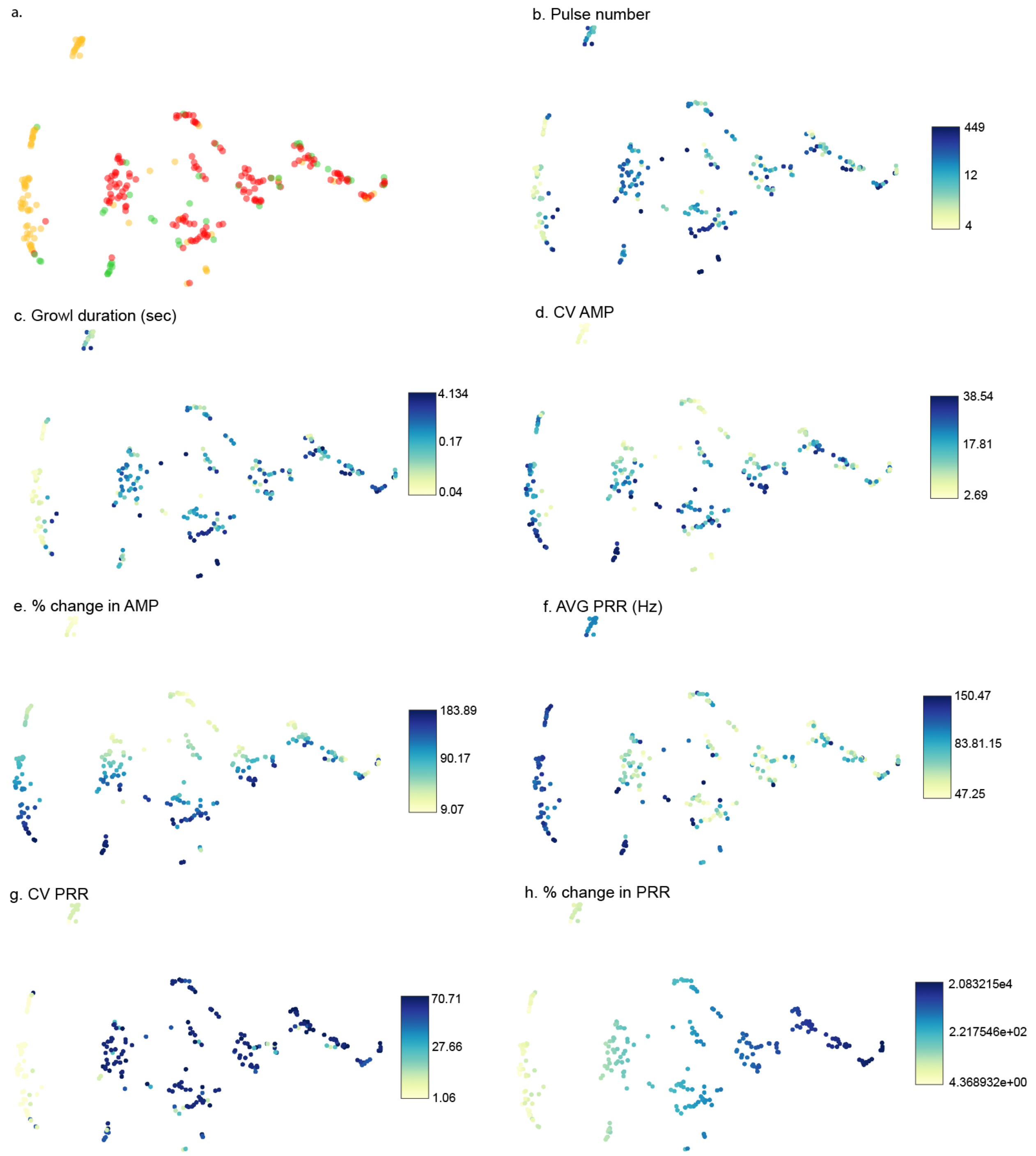

Fig. S11 Acoustic features contributing to Uniform Manifold Approximation and Projection (UMAP) cluster for glutamate (GLU)-evoked fictive growls.
Graphical breakdown showing how different acoustic features contribute to UMAP cluster for fictive growls evoked from GLU injections into the caudal superficial and deep zones of the periaqueductal gray (PAGcs and PAGcd, respectively). (**a**) UMAP plot for fictive (PAGcs, red; PAGcd, yellow) and natural (green) growls. (**b-h**) UMAP plots showing how all variables measured contribute to each cluster, including growl pulse number (**b**), duration (sec; **c**) coefficient of variation in amplitude (CV AMP, **d**), percent (%) change in AMP (**e**), average pulse repetition rate (AVG PRR, Hz; **f**), CV in PRR (Hz; **g**), %

change in PRR (Hz; **h**). In all plots yellow is the lowest value and dark blue is the highest value (see key to the right of each graph for details).

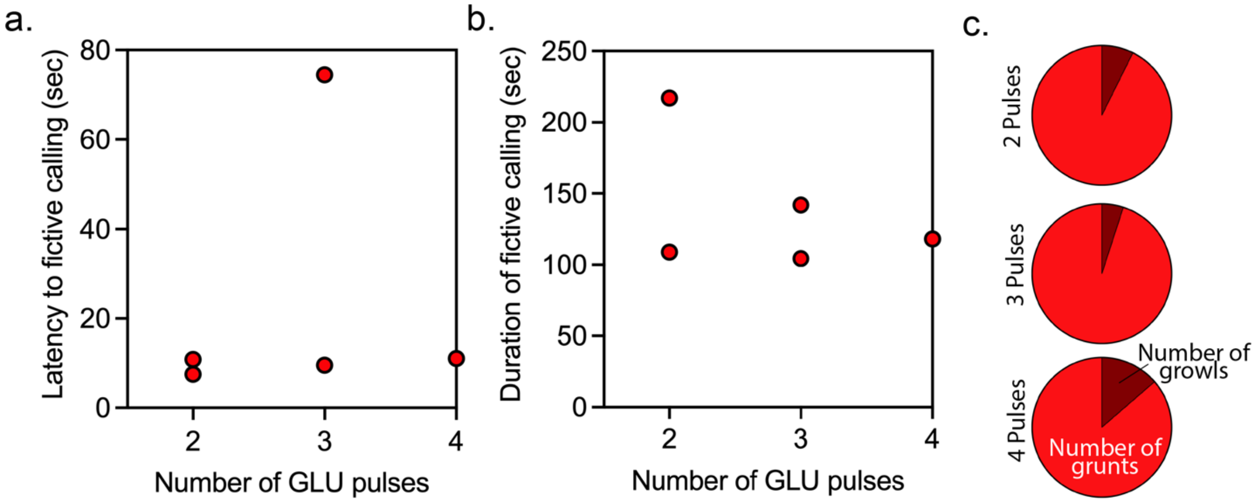

Fig. S12 Effect of glutamate (GLU) injection volume on fictive calls evoked from the caudal superficial zone of the periaqueductal gray **(PAGcs)**

(**a-c**) Increasing amounts of GLU (n = 2 animals) does not alter the latency to (**a**), duration (**b**), or types (**c**) of fictive calls evoked from PAGcs sites.

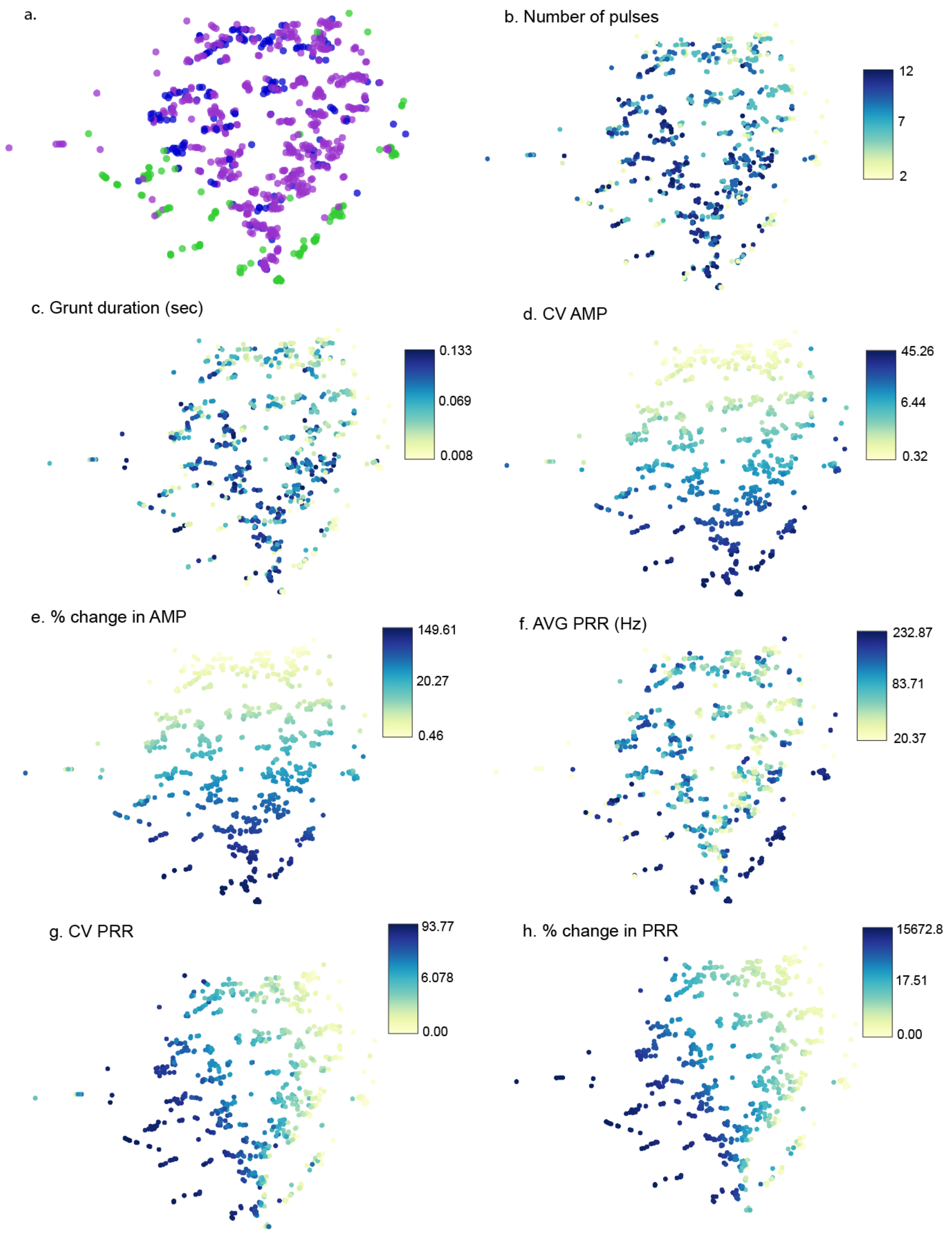

Fig. 13 Acoustic features contributing to Uniform Manifold Approximation and Projection (UMAP) for gabazine-evoked fictive grunts.
Graphical breakdown showing how different acoustic features contribute to UMAP cluster for fictive grunts evoked from gabazine injections into the caudal superficial and deep zones of the periaqueductal gray (PAGcs and PAGcd, respectively). (**a**) UMAP plot for fictive PAGcs (blue), PAGcd (purple) and natural (green) grunts. (**b-h**) UMAP plots showing how all variables measured contribute to each cluster, including grunt pulse number (**b**), duration (sec; **c**), coefficient of variation in amplitude (CV AMP, **d**), percent (%) change in AMP (**e**), average pulse repetition rate (AVG PRR, Hz; **f**), CV in PRR (Hz; **g**), %

change in PRR (Hz; **h**). In all plots yellow is the lowest value and dark blue is the highest value (see key to the right of each graph for details).

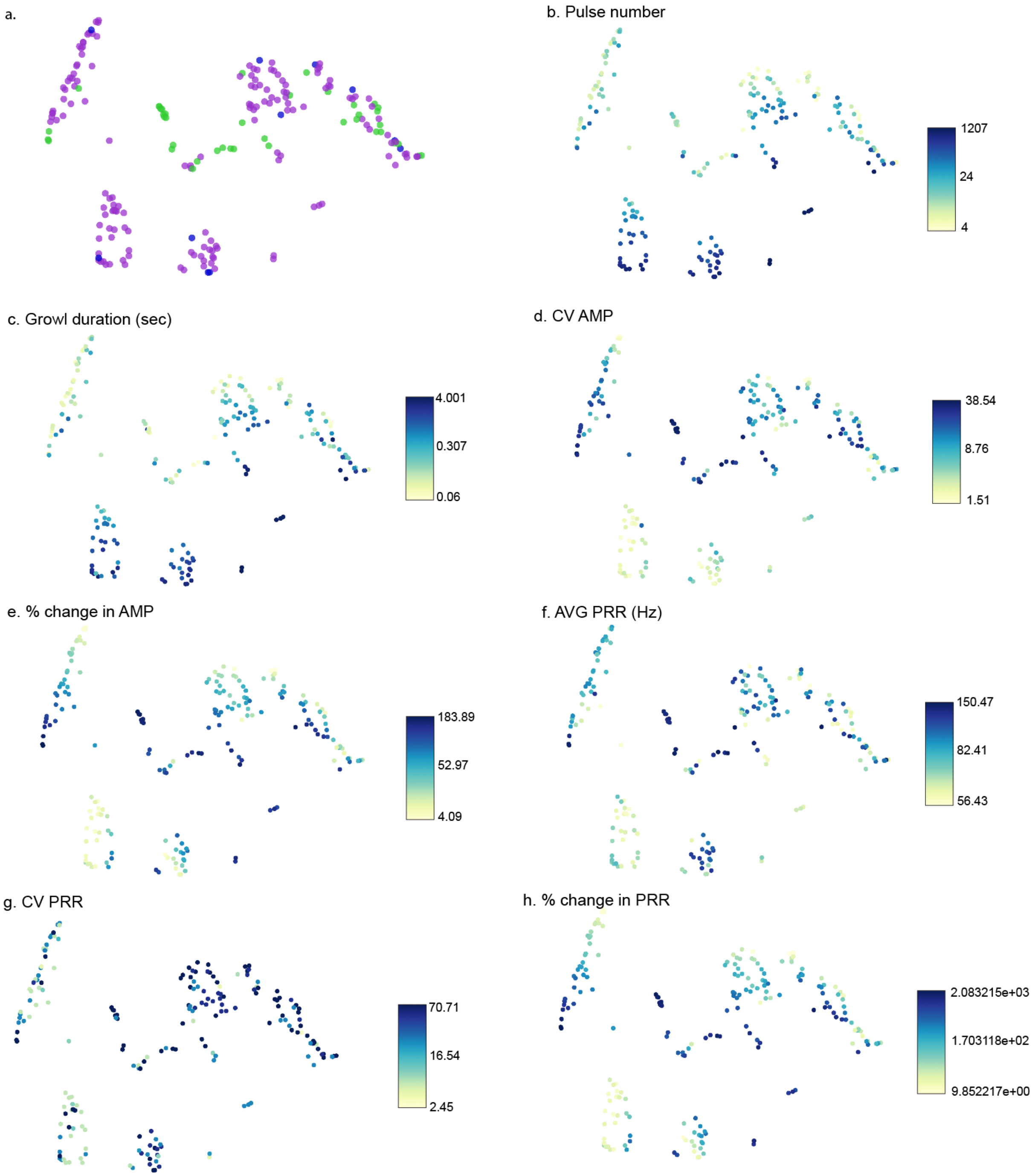

Fig. 14 Graphical breakdown of acoustic features contributing to Uniform Manifold Approximation and Projection (UMAP) cluster for gabazine-evoked fictive growls. Graphical breakdown showing how different acoustic features contribute to UMAP cluster for fictive growls evoked following gabazine injections into the caudal superficial and deep zones of the periaqueductal gray (PAGcs and PAGcd, respectively). (**a**) UMAP plot for fictive PAGcs (blue), PAGcd (purple) and natural (green) growls. (**b-h**) UMAP plots showing how all variables measured contribute to each cluster, including growl pulse number (**b**), duration (sec; **c**) and coefficient of variation in amplitude (CV AMP, **d**), percent (%) change in AMP (**e**), average pulse repetition rate (AVG PRR, Hz; **f**), coefficient of variation in PRR (Hz; **g**), % change in PRR (Hz; **h**). In all plots yellow is the lowest value and dark blue is the highest value (see key to the right of each graph for details).

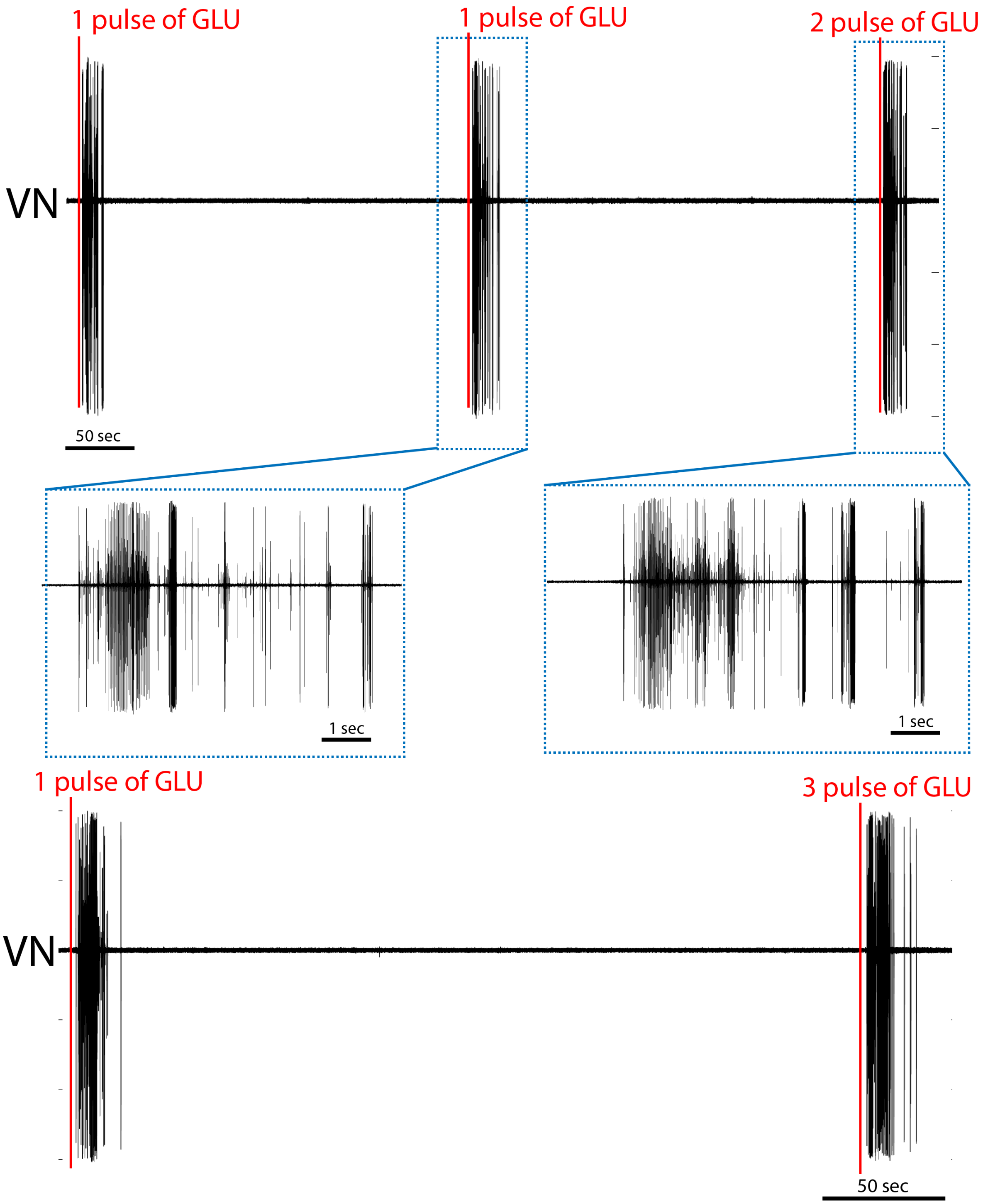

Fig. 15 Effect of glutamate (GLU) injection volume on fictive calls evoked from hindbrain vocal prepacemaker nucleus (VPP). Complete experimental fictive call traces recorded from vocal nerve (VN) of two representative male midshipman fish. Top blue hatched boxes indicate expanded lower traces of fictive calls evoked in first male from successive trials of 1 and 2 pulse GLU injections into VPP. Bottom record from second male shows fictive calls evoked from successive trials of 1 and 3 pulse GLU injections into VPP. Note that there are no noticeable differences in the duration or pattern of evoked fictive responses from each male.

Table S1. Summary of overlap between temporal features of natural calls and fictive calls evoked from caudal superficial (PAGcs) or deep (PAGcd) zones of midbrain periaqueductal gray (PAG). Bold red indicates fictive responses that are not statistically different from natural calls.

		Duration	CV PRR	CV Amplitude
**Grunt**	**Glutamate**	**PAGcs, PAGcd ~ natural**	**PAGcs ~natural**<br>PAGcd differs from natural and PAGcs	PAGcs, PAGcd differ from natural
	**Gabazine**	**PAGcs ~ natural**<br>PAGcd differs from natural and PAGcs	**PAGcs, PAGcd ~ natural**	PAGcs, PAGcd differ from natural
**Growl**	**Glutamate**	**PAGcs, PAGcd ~ natural**	**PAGcs ~ natural**<br>PAGcd differs from natural and PAGcs	**PAGcs ~ natural**<br>PAGcd differs from natural and PAGcs
	**Gabazine**	**PAGcs ~ natural**<br>PAGcd differs from natural and PAGcs	**PAGcs, PAGcd ~ natural**	PAGcs, PAGcd differ from natural

Table S2 Summary of statistics run in main text figures.

Figure #	Figure Panel and description	Statistical test	Test statistic , p value, effect size measure
**Figure 2**	i - PAGrs cfos mapping	ANOVA*	F(3,17)=1.55, p = 0.24<br>eta2=0.215
	i - PAGcs cfos mapping	ANOVA	F(3,17)=18.05, p = 1.579e-05<br>eta2=0.76
	i - PAGcd cfos mapping	ANOVA	F(3,17)=11.87, p = 0.001<br>eta2=0.677
	j - PAGcs cfos hum correlation	regression	r2=0.58, p =0.04
	k - PAGcd cfos hum correlation	regression	r2=0.55, p =0.048
	l - PAGcs cfos agonistic call correlation	regression	r2=0.76, p =0.01
	m- PAGcd cfos agonistic call correlation	regression	r2=0.35, p =0.21
**Figure 3**	f-PAGcs intron condition differences	ANOVA	F(3,16)=34.45 p = 3.272e-07<br>eta2= 0.86
	f-PAGcd intron condition differences	ANOVA	F(3,16)=20.17 p = 1.101e-05<br>eta2= 0.791
	g-PAGcs reactivation index	ANOVA	F(3,16)=81.16 p = 6.782e-10<br>eta2= 0.938
	g-PAGcd reactivation index	ANOVA	F(3,16)=39.51 p= 1.262e-07<br>eta2= 0.881
	r-PAGcs intron condition differences	t-test	t(7)=2.68, p=0.03<br>cohen's d=1.84
	r-PAGcd intron condition differences	t-test	t(7)=3.38, p=0.014<br>cohen's d=2.24
	s-PAGcs reactivation index feeding	t-test	t(7)=9.79, p<0.001<br>cohen's d=1.58
	s-PAGcd reactivation index feeding	t-test	t(7)=1.57, p=0.17<br>cohen's d=1.11
**Figure 4**	e; grunt duration	LMM*	F(2,22.36)=0.98, p=0.39<br>eta2=0.14
	f; grunt CV PRR	LMM	F(2,16.03)=4.51, p=0.02<br>eta2=0.36
	g; grunt CV AMP	LMM	F(2,21.53)=11.85, p=0.0003<br>eta2=0.52
	h; growl duration	LMM	F(2,8.68)=0.699, p=0.522<br>eta2=0.14
	I; growl CV PRR	LMM	F(2,9.89)=7.92, p=0.008<br>eta2=0.62
	j; growl CV AMP	LMM	F(2,13.75)=6.27, p=0.011<br>eta2=0.48
**Figure 5**	c; grunt duration	LMM	F(2,8.63)=3.87, p=0.02<br>eta2=0.47
	d; grunt CV PRR	LMM	F(2,3.60)=1.21, p=0.40<br>eta2=0.4
	e; grunt CV AMP	LMM	F(2,6.71)=24.398, p=0.00083<br>eta2=0.88

	f; growl duration	LMM	F(2,9.47)=4.306, p=0.04<br>eta2=0.48
	g; growl CV PRR	LMM	F(2,5.79)=0.54, p=0.61<br>eta2=0.16
	h; growl CV AMP	LMM	F(2,6.11)=30.35, p=0.00067<br>eta2=0.91
**Figure 6**	d- PAG vs VPP latency	t-test	t(5)=2.74 , p=0.04
	e- PAG vs VPP Duration	t-test	t(5)=9.06 , p=0.0003
	k; grunt duration	LMM	F(1,6.82)=0.57, p=0.47<br>eta2=0.08
	l; grunt CV PRR	LMM	F(1,5.59)=0.32, p=0.59<br>eta2=0.05
	m; grunt CV AMP	LMM	F(1,7.11)=0.857, p=0.38<br>eta2=0.11
	n; growl duration	LMM	F(1,5.818)=5.68, p=0.01<br>eta2=0.49
	o; growl CV PRR	LMM	F(1,8.78)=4.383, p=0.06<br>eta2=0.33
	p; growl CV AMP	LMM	F(1,2.23)=2.13, p=0.17<br>eta2=0.20

* ANOVA = Analysis of variance and LMM = linear mixed model. In all linear mixed models, animal ID was used as a random effect.

Movie S1. Example of male plainfin midshipman (*Porichthys notatus*) courtship humming in a laboratory setting. Video taken at nighttime under red-light conditions illustrates a male humming from inside his artificial nest. Unlike a male that is producing agonistic vocalizations (see Movie S2), humming males inflate their swim bladder during humming[9]. A consequence of inflating the swim bladder is that the male becomes positively buoyant; the terracotta top of the nest keeps him from floating to the surface of the tank. Note that the video only contains the near constant frequency portion of the hum, and not the hum onset (see Figs. 1a, 5a). Video credit: Margaret A. Marchaterre.

Movie S2. Example of simulated territorial intrusion with 3D printed model male plainfin midshipman (*Porichthys notatus*). Video taken at "night" under red-light conditions illustrates nesting male midshipman grunting, growling, and lunging while being chased by a 3D printed model of a midshipman male. Terracotta nest top removed for visual clarity in video. Video credit: Eric Schuppe and Margaret A. Marchaterre.

Movie S3. Example of Midshipman feeding on a goldfish during catFISH trials. Video taken at "night" under red-light conditions illustrates male midshipman eating a goldfish that swam into its nest.

Supplementary References:

1. Kittelberger, J. M. & Bass, A. H. Vocal-motor and auditory connectivity of the midbrain periaqueductal gray in a teleost fish. *Journal of Comparative Neurology* **521**, 791–812 (2013).
2. Kittelberger, M. J., Land, B. R. & Bass, A. H. Midbrain periaqueductal gray and vocal patterning in a teleost fish. *J Neurophysiol* **96**, 71–85 (2006).
3. Goodson, J. L. & Bass, A. H. Vocal-acoustic circuitry and descending vocal pathways in teleost fish: Convergence with terrestrial vertebrates reveals conserved traits. *Journal of Comparative Neurology* **448**, 298–322 (2002).
4. Chagnaud, B. P., Baker, R. & Bass, A. H. Vocalization frequency and duration are coded in separate hindbrain nuclei. *Nat Commun* **2**, (2011).
5. Bass, A. H., Bodnar, D. A. & Marchaterre, M. A. Midbrain acoustic circuitry in a vocalizing fish. *Journal of Comparative Neurology* **419**, 505–531 (2000).
6. Bass, A. H., Gilland, E. H. & Baker, R. Evolutionary origins for social vocalization in a vertebrate hindbrain-spinal compartment. *Science (1979)* **321**, 417–421 (2008).
7. Bass, A. H. & Baker, R. Sexual dimorphisms in the vocal control system of a teleost fish: Morphology of physiologically identified neurons. *J Neurobiol* **21**, 1155–1168 (1990).
8. Rubow, T. K. & Bass, A. H. Reproductive and diurnal rhythms regulate vocal motor plasticity in a teleost fish. *Journal of Experimental Biology* **212**, 3252–3262 (2009).
9. Bass, A. H., Chagnaud, B. P. & Feng, N. Y. Comparative Neurobiology of Sound Production in Fishes. in *Comparative Neurobiology of Sound Production in Fishes* 35–75 (2015). doi:10.1007/978-3-7091-1846-7_2.
