# Supplementary material for: Midbrain node for context-specific vocalisation in fish
Source: Nat Commun. 2024 Jan 2;15:189. doi: 10.1038/s41467-023-43794-y (PMC10762186; doi:10.1038/s41467-023-43794-y)
Supplement: Supplementary file 3 — Description of Additional Supplementary Files [file 41467_2023_43794_MOESM3_ESM.pdf]

Description of Additional Supplementary Files

File Name: Movie S1
Description: Video taken at nighttime under red-light conditions illustrates a male humming from inside his artificial nest. Unlike a male that is producing agonistic vocalizations (see Movie S2), humming males inflate their swim bladder during humming9. A consequence of inflating the swim bladder is that the male becomes positively buoyant; the terracotta top of the nest keeps him from floating to the surface of the tank. Note that the video only contains the near constant frequency portion of the hum, and not the hum onset (see Figs. 1a, 5a). Video credit: Margaret A. Marchaterre.

File Name: Movie S2
Description: Video taken at "night" under red-light conditions illustrates nesting male midshipman grunting, growling, and lunging while being chased by a 3D printed model of a midshipman male. Terracotta nest top removed for visual clarity in video. Video credit: Eric Schuppe and Margaret A. Marchaterre.

File Name: Movie S3
Description: Video taken at "night" under red-light conditions illustrates male midshipman eating a goldfish that swam into its nest.
