# Supplementary material for: Midbrain node for context-specific vocalisation in fish
Source: Nat Commun. 2024 Jan 2;15:189. doi: 10.1038/s41467-023-43794-y (PMC10762186; doi:10.1038/s41467-023-43794-y)
Supplement: Supplementary file 7 — Reporting Summary [file 41467_2023_43794_MOESM7_ESM.pdf]

nature portfolio

Corresponding author(s): Eric Schuppe / Andrew H. Bass

Last updated by author(s): Feb 13, 2023

Reporting Summary

Nature Portfolio wishes to improve the reproducibility of the work that we publish. This form provides structure for consistency and transparency in reporting. For further information on Nature Portfolio policies, see our Editorial Policies and the Editorial Policy Checklist.

Statistics

For all statistical analyses, confirm that the following items are present in the figure legend, table legend, main text, or Methods section.

n/a	Confirmed	
☐	☒	The exact sample size (*n*) for each experimental group/condition, given as a discrete number and unit of measurement
☐	☒	A statement on whether measurements were taken from distinct samples or whether the same sample was measured repeatedly
☐	☒	The statistical test(s) used AND whether they are one- or two-sided<br>*Only common tests should be described solely by name; describe more complex techniques in the Methods section.*
☒	☐	A description of all covariates tested
☐	☒	A description of any assumptions or corrections, such as tests of normality and adjustment for multiple comparisons
☐	☒	A full description of the statistical parameters including central tendency (e.g. means) or other basic estimates (e.g. regression coefficient) AND variation (e.g. standard deviation) or associated estimates of uncertainty (e.g. confidence intervals)
☐	☒	For null hypothesis testing, the test statistic (e.g. *F*, *t*, *r*) with confidence intervals, effect sizes, degrees of freedom and *P* value noted<br>*Give P values as exact values whenever suitable.*
☒	☐	For Bayesian analysis, information on the choice of priors and Markov chain Monte Carlo settings
☒	☐	For hierarchical and complex designs, identification of the appropriate level for tests and full reporting of outcomes
☐	☒	Estimates of effect sizes (e.g. Cohen's *d*, Pearson's *r*), indicating how they were calculated

Our web collection on statistics for biologists contains articles on many of the points above.

Software and code

Policy information about availability of computer code

Data collection	For our behavioral assays, audio files were saved in audacity. To remotely monitor humming, we used TeamViewer. All physiology recordings were recorded in Clampfit 9. All in situ and tracing images were acquired using the Zen software on a Zeiss confocal. All image analysis was performed using imageJ by a blind observer. No custom scripts were used for data acquisition
Data analysis	All data analysis was performed in R v3.3.2 or MATLAB 2021a. All custom scripts to analyze sound and neurophysiology data will be made available upon request.

For manuscripts utilizing custom algorithms or software that are central to the research but not yet described in published literature, software must be made available to editors and reviewers. We strongly encourage code deposition in a community repository (e.g. GitHub). See the Nature Portfolio guidelines for submitting code & software for further information.

Data

Policy information about availability of data

All manuscripts must include a data availability statement. This statement should provide the following information, where applicable:

- Accession codes, unique identifiers, or web links for publicly available datasets
- A description of any restrictions on data availability
- For clinical datasets or third party data, please ensure that the statement adheres to our policy

All data are included as part of the supplemental materials

Human research participants

Policy information about studies involving human research participants and Sex and Gender in Research.

Reporting on sex and gender	N/A
Population characteristics	N/A
Recruitment	N/A
Ethics oversight	N/A

Note that full information on the approval of the study protocol must also be provided in the manuscript.

Field-specific reporting

Please select the one below that is the best fit for your research. If you are not sure, read the appropriate sections before making your selection.

☒ Life sciences ☐ Behavioural & social sciences ☐ Ecological, evolutionary & environmental sciences

For a reference copy of the document with all sections, see nature.com/documents/nr-reporting-summary-flat.pdf

Life sciences study design

All studies must disclose on these points even when the disclosure is negative.

Sample size	In total, we used 111 in fish across 10 unique experiments. Each of these experiments, often had between 3-4 behavioral treatment groups. In most cases, we tried to have a minimum sample size of 5 animals per treatment group. No sample size calculation was performed at the onset of experiments. Instead, this our sample size numbers for each experiment were chosen based on existing standards in the field. At the same time, this number was chosen to achieve statistical robustness, but also keep in mind the difficultly of performing some of these behavioral studies on a limited number of wild caught fish.
Data exclusions	No animals were excluded from data analysis
Replication	All experiments included multiple replicates of individually housed animals. All cell counts from in situ hybridization were analyzed by a blind observer and a trained neural network (cellpose 2 A second blind observer repeated 40 random images. This approached ensured the accuracy and validity of cell counts.
Randomization	For all behavioral studies, animals were picked at random. When possible in our behavioral analyses, we randomly assigned animals to groups.
Blinding	In cases, where there was cell counting across treatment groups an observer that was blind to the condition performed such analyses.

Reporting for specific materials, systems and methods

We require information from authors about some types of materials, experimental systems and methods used in many studies. Here, indicate whether each material, system or method listed is relevant to your study. If you are not sure if a list item applies to your research, read the appropriate section before selecting a response.

Materials & experimental systems

n/a	Involved in the study	
☐	☒	Antibodies
☒	☐	Eukaryotic cell lines
☒	☐	Palaeontology and archaeology
☐	☒	Animals and other organisms
☒	☐	Clinical data
☒	☐	Dual use research of concern

Methods

n/a	Involved in the study	
☒	☐	ChIP-seq
☒	☐	Flow cytometry
☒	☐	MRI-based neuroimaging

Antibodies

Antibodies used	Anti-GABA (Synaptic systems;1:500); Anti-GLU (Sigma-Aldrich; G6642;1:500); PSD95 (mouse monoclonal, Millipore, MAB1596) ; vglut1/2 (rabbit polyclonal, synaptic systems: AB135 503

Validation	Previous experiments have extensively validated both antibodies. For instance, the GLU antibody specifically labels neurons expressing a fluorescent protein under control of the vglut2 promoter, and preadsorption with glutamate eliminates labeling in another teleost. Likewise, with the GABA antibody, elimination of label following preadsorption of the antibody with GABA has been reported in a broad range of species (see method section for details). The vglut1/2 antibody has been previously validated for use in teleost species. Finally, the PSD95 antibody was chosen because it had been validated across vertebrates species, including teleost fish

Animals and other research organisms

Policy information about studies involving animals; ARRIVE guidelines recommended for reporting animal research, and Sex and Gender in Research

Laboratory animals	No lab animals were used in this study.
Wild animals	All adult male plainfin midshipman fish were caught near the UC Davis Bodega marine lab (California,USA). After being caught, animals heath and body condition was screened. Healthy animals were immediately shipped overnight to our lab at Cornell University. When they arrived there, they were housed individually
Reporting on sex	Only males were used in these experiments. Females in this species do not produce complex courtship vocalizations or actively defend nests in the same way as males.
Field-collected samples	Field collected adult male midshipman were used in this study (see above).
Ethics oversight	The capture of midshipman fish was approved by CA Fish and game with the assistance of personnel at the UC Davis Marine lab. All animal work at Cornell was approved by the IACUC (#2005-0118)

Note that full information on the approval of the study protocol must also be provided in the manuscript.
